# Quality of Life and Health Awareness Among Patients With Hypertension in Urban and Rural Regions of Riyadh City, Saudi Arabia

**DOI:** 10.7759/cureus.72178

**Published:** 2024-10-23

**Authors:** Lamia K Albugami, Ashwag Alharbi, Hind A Alotaibi, Elaf mohmmad, Aseel S Altoom, Ebtesam H alotaibi

**Affiliations:** 1 Nursing, National Guard Health Affairs, Riyadh, SAU

**Keywords:** health awareness, health-related quality of life, hypertension, saudi arabia, urban and rural disparities

## Abstract

Background: Hypertension is a leading global health issue and a major risk factor for cardiovascular diseases. Understanding the differences in quality of life (QoL) and health awareness among hypertensive patients in urban and rural regions is critical for developing targeted interventions to improve health outcomes. This study aims to assess the health-related quality of life (HRQoL) and health awareness among hypertensive patients in Riyadh, Saudi Arabia.

Methods: A cross-sectional design was adopted to evaluate 246 hypertensive patients attending public and private hospitals in Riyadh. Data were collected using structured questionnaires covering socio-demographics, HRQoL (measured via the SF-36), and health awareness. Descriptive statistics, Pearson correlation coefficients, and inferential analysis were performed using SPSS version 26 (IBM Corp., Armonk, NY), with significance set at p < 0.05.

Results: The mean age of participants was 55.2 years (SD = 10.3), with 55.7% being male. The overall HRQoL score was 62.7 (SD = 17.3), reflecting a moderate level of quality of life. Emotional well-being scored the highest (72.2, SD = 16.4), while energy/fatigue scored the lowest (55.4, SD = 14.9). Health awareness levels were high regarding the importance of blood pressure monitoring (mean = 4.7, SD = 0.5) and lifestyle changes (mean = 4.6, SD = 0.5), but moderate for stress management (mean = 4.1, SD = 0.7). Significant positive correlations were found between QoL and awareness of regular blood pressure monitoring (r = 0.50, p < 0.001) and lifestyle changes (r = 0.48, p < 0.001).

Conclusion: The study revealed a moderate level of HRQoL among hypertensive patients in Riyadh, with notable gaps in awareness of stress management and other lifestyle factors. Tailored health education programs focusing on these areas are essential to improving hypertension management and enhancing overall quality of life, particularly in rural populations with limited healthcare access.

## Introduction

Hypertension is one of the most prevalent chronic diseases worldwide, affecting millions of individuals in both urban and rural settings. It is a major risk factor for cardiovascular diseases, stroke, and kidney failure. Managing hypertension effectively is crucial to improving patients' health outcomes and quality of life (QoL). Despite significant progress in understanding hypertension, there are still considerable differences in how it affects individuals living in urban versus rural areas. These differences may stem from various factors, such as access to healthcare, socio-economic status, and health literacy [1]. Exploring these disparities is essential to developing targeted interventions that improve the quality of life for hypertensive patients, particularly those in underserved rural areas.

The quality of life for hypertensive patients is closely linked to various determinants, including the availability of healthcare services, the level of health education, and patients' adherence to treatment protocols. Research has shown that individuals in urban areas typically have better access to healthcare services and resources, leading to improved management of their condition and a higher quality of life [2]. In contrast, rural populations often face numerous barriers, such as limited healthcare infrastructure and a lack of specialized services, which can result in poorer health outcomes and a lower quality of life. In addition, socio-economic factors, including education and income, play a significant role in how individuals manage their hypertension and their overall well-being [3].

Several studies have examined the specific factors influencing the quality of life among hypertensive individuals in rural areas. For instance, a cross-sectional study conducted in rural China revealed that the health-related quality of life (HRQoL) of hypertensive patients was significantly lower than that of their urban counterparts, largely due to inadequate healthcare access and poor adherence to medication regimens [4]. The study highlighted the need for public health interventions focused on improving health education and providing rural residents easier access to healthcare services. Additionally, informal caregivers in rural areas often lack the necessary knowledge and resources to provide adequate care, further exacerbating the challenges faced by hypertensive patients [5].

Urban environments, on the other hand, offer more robust healthcare infrastructures, which tend to improve hypertension management and patient outcomes. Urban residents generally have higher health awareness, access to educational programs, and better healthcare services, all of which contribute to better control of hypertension and, ultimately, a higher quality of life [6]. However, this does not mean that urban populations are immune to challenges. Even in urban settings, certain socio-economic factors, such as income inequality and access to affordable healthcare, can negatively impact the management of hypertension and the quality of life [7].

One important aspect that influences the quality of life among hypertensive patients is their self-perception of aging and health. Studies suggest that urban hypertensive patients often have a more positive perception of their aging process and health status, which correlates with better health-related quality of life outcomes [8]. In contrast, rural patients tend to have lower perceptions of their health, which may be due to the higher prevalence of comorbidities and the lack of resources to manage their condition effectively [9]. This highlights the importance of psychological and social support as part of the comprehensive management of hypertension, particularly in rural areas where such resources are often lacking.

While hypertension is a global health issue, the impact of the disease on patients' quality of life varies significantly between urban and rural populations. Factors such as healthcare access, socio-economic status, health education, and self-perception of health all contribute to these disparities. Addressing the unique challenges faced by rural hypertensive patients, including improving healthcare infrastructure and providing targeted health education programs, is essential to reducing these inequalities and improving the overall quality of life for this population [10].

## Materials and methods

Research design

This study adopted a cross-sectional research design to assess the quality of life and health awareness among hypertensive patients attending both public and private hospitals in Riyadh, Saudi Arabia. A cross-sectional design was selected as it allows for data collection at a single point in time, which is appropriate for understanding the relationships between HRQoL and various influencing factors, including demographics, awareness, and healthcare access.

Research population and sample

The research population consists of hypertensive patients attending public and private hospitals in Riyadh, Saudi Arabia. Given the inclusion of both public and private healthcare settings, the study captures a diverse range of socioeconomic backgrounds and healthcare access levels, providing a comprehensive understanding of the population. The sample size was calculated using the Raosoft sample size calculator with the assumption of an unknown population size, a confidence level of 95%, a margin of error of 5%, and a response distribution of 50%. Based on these parameters, the required sample size was determined to be 246 patients. These patients were selected using convenience sampling from multiple hospitals in Riyadh to ensure a diverse sample that represents the broader population of hypertensive patients in the city.

Data collection tools

Data were collected using a structured questionnaire that comprised three key sections: demographic information, HRQoL assessment, and health awareness assessment.

Demographic Information

This section collected data on participants' age, gender, education level, employment status, duration of hypertension, and type of hospital attended (public or private).

HRQoL Assessment

The HRQoL was measured using the validated and reliable SF-36 (Short Form Health Survey). This tool evaluates eight health domains: physical functioning, role limitations due to physical health, role limitations due to emotional problems, energy/fatigue, emotional well-being, social functioning, pain, and general health. The SF-36 has been widely used in similar studies and is known for its strong validity and reliability. The internal consistency of the SF-36 has been previously reported to have Cronbach's alpha values ranging from 0.77 to 0.93 across its subscales, indicating excellent reliability. Scores for each domain range from 0 to 100, with higher scores indicating better health-related quality of life.

Health Awareness Assessment

A self-developed questionnaire was used to measure participants' awareness of hypertension-related risk factors, complications, and the importance of lifestyle changes. The questionnaire was developed based on a literature review and consultations with subject-matter experts. It was pretested with a small subset of patients to ensure clarity and face validity. The final version of the questionnaire contained 15 items using a Likert scale format (1 = strongly disagree, 5 = strongly agree) to gauge awareness. Content validity was ensured through expert review, and the tool demonstrated good reliability, with a Cronbach's alpha of 0.81 in the pilot test.

Data collection procedure

Data collection was conducted over a period of two months from patients attending outpatient clinics in public and private hospitals in Riyadh. Trained research assistants approached eligible hypertensive patients, explained the study's purpose, and obtained informed consent. Participants who agreed to participate were given the questionnaire to complete on-site. Research assistants were available to answer questions and clarify items if needed to maintain consistency and minimize response bias. Participants were assured of the confidentiality of their responses, and no personally identifiable information was collected. The average time to complete the questionnaire was approximately 20 minutes.

Ethical considerations

Ethical approval for this study was obtained from the relevant bodies. All participants were fully informed about the nature and purpose of the study, and their participation was voluntary. Informed consent was obtained from each participant before any data were collected. Participants were assured that their information would be kept confidential and that they could withdraw from the study at any time without any repercussions. The study was conducted in accordance with the ethical principles outlined in the Declaration of Helsinki.

Data analysis

Data were analyzed using SPSS (Statistical Package for the Social Sciences, IBM Corp., Armonk, NY) version 26. Descriptive statistics, including frequencies, percentages, means, and standard deviations, were used to summarize demographic characteristics and the overall health-related quality of life and awareness scores. Pearson correlation coefficients were calculated to examine the relationships between demographic variables, health awareness, and HRQoL. Inferential statistics assessed the strength and direction of associations between variables. A significance level of alpha (α) < 0.05 was set as the threshold for statistical significance.

## Results

Baseline socio-demographic characteristics

Table 1 presents the baseline socio-demographic characteristics of the 246 hypertensive patients. The mean age of the participants was 55.2 years (SD = 10.3). Most patients were male (55.7%, n = 137), with females comprising 44.3% (n = 109) of the sample. Regarding educational attainment, 13% (n = 32) of the participants were illiterate, 19.1% (n = 47) had completed primary education, 30.5% (n = 75) had completed secondary education, and 37.4% (n = 92) had attained higher education. In terms of employment, 44.7% (n = 110) were employed, while 55.3% (n = 136) were unemployed. Most participants received care at public hospitals (57.7%, n = 142), while the remainder attended private hospitals (42.3%, n = 104).

**Table 1:** Baseline socio-demographic characteristics of the enrolled hypertensive patients (n=246)

Variable	Frequency (%)
Age (years) - mean (SD)	55.2 (±10.3)
Gender	
Male	137 (55.7%)
Female	109 (44.3%)
Education	
Illiterate	32 (13%)
Primary	47 (19.1%)
Secondary	75 (30.5%)
Higher	92 (37.4%)
Employment	
Employed	110 (44.7%)
Unemployed	136 (55.3%)
Hospital type	
Public	142 (57.7%)
Private	104 (42.3%)

The levels of health-related quality of life

The HRQoL of the participants, as measured by the SF-36, is summarized in Table 2. The overall mean HRQoL score was 62.7 (SD = 17.3), indicating a moderate level of quality of life among the hypertensive patients. Physical functioning scored a mean of 68.5 (SD = 15.3), which was classified as moderate, while role limitations due to physical health had a mean score of 62.1 (SD = 20.2), which was also rated as moderate. Similarly, the domain of role limitations due to emotional problems had a moderate rating, with a mean score of 59.3 (SD = 18.7).

Energy/fatigue had the lowest score, with a mean of 55.4 (SD = 14.9), indicating a low level in this domain. In contrast, emotional well-being had the highest score of 72.2 (SD = 16.4), classified as high. The social functioning domain had a mean score of 64.7 (SD = 17.2), which was rated as moderate. The pain domain and general health both had moderate scores, with means of 60.5 (SD = 16.3) and 58.9 (SD = 19.5), respectively. Overall, these results suggest a generally moderate level of health-related quality of life across most domains, except for energy/fatigue, which was low, and emotional well-being, which was high.

**Table 2:** Participants’ health-related quality of life (HRQoL) responses with mean scores, standard deviations, and levels based on the SF-36 questionnaire

Health domain	Mean score (SD)	Level
Physical functioning	68.5 (±15.3)	Moderate
Role limitations due to physical health	62.1 (±20.2)	Moderate
Role limitations due to emotional problems	59.3 (±18.7)	Moderate
Energy/fatigue	55.4 (±14.9)	Low
Emotional well-being	72.2 (±16.4)	High
Social functioning	64.7 (±17.2)	Moderate
Pain	60.5 (±16.3)	Moderate
General health	58.9 (±19.5)	Moderate
Total QoL	62.7 (±17.3)	Moderate

Health awareness regarding hypertension-related factors

Table 3 presents the participants' health awareness regarding factors related to hypertension. The overall awareness of hypertension as a serious health condition was high, with a mean score of 4.5 (SD = 0.7). Participants demonstrated a high level of awareness in several areas, including the importance of regular blood pressure monitoring (M = 4.7, SD = 0.5), understanding of complications such as stroke and heart disease (M = 4.4, SD = 0.6), and awareness of lifestyle changes as part of treatment (M = 4.6, SD = 0.5).

Moderate awareness was reported in areas such as knowledge of risk factors (e.g., obesity, smoking) (M = 4.2, SD = 0.8), medication adherence (M = 4.3, SD = 0.7), and the role of physical activity in managing hypertension (M = 4.3, SD = 0.6). Participants had moderate knowledge about reducing salt intake (M = 4.1, SD = 0.8), weight management (M = 4.0, SD = 0.9), and alcohol consumption's effect on blood pressure (M = 3.9, SD = 0.8).

Further, understanding of stress management (M = 4.1, SD = 0.7) and hereditary risk factors for hypertension (M = 4.0, SD = 0.8) were also rated as moderate. Participants demonstrated a high level of awareness regarding the need for regular healthcare visits (M = 4.5, SD = 0.6). Awareness of the benefits of smoking cessation (M = 4.2, SD = 0.7) and the relationship between hypertension and mental health (M = 4.1, SD = 0.8) were also rated as moderate. Overall, participants showed a high level of awareness in some key areas but moderate levels in others, highlighting potential areas for targeted health education.

**Table 3:** Participants’ health awareness assessment: mean scores, standard deviations, and levels for hypertension-related awareness

Questionnaire Item	Mean score (SD)	Level
Awareness of hypertension as a serious health condition	4.5 (±0.7)	High
Knowledge of risk factors (e.g., obesity, smoking)	4.2 (±0.8)	Moderate
Awareness of the importance of regular blood pressure monitoring	4.7 (±0.5)	High
Understanding of the complications (e.g., stroke, heart disease)	4.4 (±0.6)	High
Awareness of lifestyle changes (e.g., diet, exercise) as treatment	4.6 (±0.5)	High
Awareness of medication adherence and its impact on hypertension	4.3 (±0.7)	Moderate
Knowledge about reducing salt intake	4.1 (±0.8)	Moderate
Understanding the importance of weight management	4.0 (±0.9)	Moderate
Awareness of the role of physical activity in managing hypertension	4.3 (±0.6)	Moderate
Awareness of alcohol consumption's effect on blood pressure	3.9 (±0.8)	Moderate
Understanding stress management and its impact on hypertension	4.1 (±0.7)	Moderate
Knowledge of hereditary risk factors for hypertension	4.0 (±0.8)	Moderate
Awareness of the need for regular healthcare visits	4.5 (±0.6)	High
Understanding the benefits of smoking cessation for hypertension	4.2 (±0.7)	Moderate
Awareness of the relationship between hypertension and mental health	4.1 (±0.8)	Moderate

The association between health-related quality of life and awareness levels

Table 4 presents the association between total QoL and the 15 health awareness domains. A significant positive correlation was found between total QoL and awareness of hypertension as a serious health condition (r = 0.45, p < 0.001), as well as knowledge of risk factors (r = 0.38, p < 0.001). The strongest association was observed between total QoL and awareness of the importance of regular blood pressure monitoring (r = 0.50, p < 0.001). Understanding the complications of hypertension (r = 0.42, p < 0.001) and awareness of lifestyle changes (r = 0.48, p < 0.001) were also significantly correlated with QoL.

Additional significant associations were found for awareness of medication adherence (r = 0.40, p < 0.001), knowledge about reducing salt intake (r = 0.37, p < 0.001), and understanding the importance of weight management (r = 0.36, p < 0.001). Awareness of physical activity's role in managing hypertension (r = 0.43, p < 0.001) also strongly correlated with QoL.

Weaker but still significant correlations were found for awareness of alcohol consumption's effect on blood pressure (r = 0.31, p = 0.002), stress management (r = 0.35, p = 0.001), and hereditary risk factors for hypertension (r = 0.33, p = 0.002). Additionally, significant correlations were observed for awareness of the need for regular healthcare visits (r = 0.46, p < 0.001), understanding the benefits of smoking cessation (r = 0.39, p < 0.001), and awareness of the relationship between hypertension and mental health (r = 0.34, p = 0.001).

**Table 4:** Association between total quality of life and health awareness domains

Health awareness domain (15 items)	Pearson correlation with total QoL	Significance (p-value)
Awareness of hypertension as a serious health condition	0.45	<0.001
Knowledge of risk factors (e.g., obesity, smoking)	0.38	<0.001
Awareness of the importance of regular blood pressure monitoring	0.50	<0.001
Understanding of the complications (e.g., stroke, heart disease)	0.42	<0.001
Awareness of lifestyle changes (e.g., diet, exercise) as treatment	0.48	<0.001
Awareness of medication adherence and its impact on hypertension	0.40	<0.001
Knowledge about reducing salt intake	0.37	<0.001
Understanding the importance of weight management	0.36	<0.001
Awareness of the role of physical activity in managing hypertension	0.43	<0.001
Awareness of alcohol consumption's effect on blood pressure	0.31	0.002
Understanding stress management and its impact on hypertension	0.35	0.001
Knowledge of hereditary risk factors for hypertension	0.33	0.002
Awareness of the need for regular healthcare visits	0.46	<0.001
Understanding the benefits of smoking cessation for hypertension	0.39	<0.001
Awareness of the relationship between hypertension and mental health	0.34	0.001

## Discussion

This study aims to assess the HRQoL and health awareness among hypertensive patients in Riyadh, Saudi Arabia. The findings of this study provide significant insights into the HRQoL and health awareness among hypertensive patients in Riyadh, as well as the association between these two variables. The mean HRQoL score of 62.7 (SD = 17.3), classified as moderate, highlights that hypertension can substantially affect individuals' well-being, particularly in domains such as energy/fatigue and general health, where participants reported lower scores. This is consistent with previous studies that emphasize how chronic conditions, such as hypertension, are associated with reduced HRQoL due to their impact on physical and emotional functioning [8]. Hypertension-related complications, including the increased risk of stroke and heart disease, can severely affect patients' overall well-being, contributing to the moderate HRQoL reported in this study [9].

One of the key findings was the high awareness of hypertension as a serious health condition, along with awareness of the importance of regular blood pressure monitoring and lifestyle changes, such as diet and exercise, as part of treatment. This reflects a strong level of health literacy among participants, which is crucial in managing chronic conditions like hypertension. Studies have shown that greater awareness of hypertension is associated with better disease management and improved health outcomes, as patients are more likely to adhere to treatment and lifestyle modifications [10]. However, despite the high awareness in some areas, other domains, such as awareness of alcohol consumption's effect on blood pressure and stress management, showed moderate scores, indicating potential areas for health education interventions.

Interestingly, the study found that certain domains of health awareness, such as the importance of regular blood pressure monitoring and lifestyle changes, had a strong positive correlation with total QoL. This aligns with previous research indicating that health-promoting behaviors, such as regular exercise, a healthy diet, and routine monitoring, are significant contributors to better HRQoL among hypertensive patients [11]. For instance, adopting a health-promoting lifestyle has been found to be a key factor in improving the quality of life for patients with hypertension [12]. These behaviors not only help control blood pressure but also enhance physical and emotional well-being, further supporting the findings of this study.

Conversely, the lower levels of awareness regarding the impact of alcohol consumption and stress management on hypertension highlight gaps in patients' understanding of the full range of risk factors associated with the condition. Stress is a well-known contributor to increased blood pressure and hypertension-related complications, yet awareness of its impact was rated as moderate in this study. This is concerning, given the evidence linking stress management with better hypertension control and overall HRQoL [9]. Targeted education on stress reduction techniques, such as mindfulness and relaxation exercises, could benefit this population, as previous research has shown that these interventions can significantly improve HRQoL [8].

The study also found significant associations between knowledge of risk factors, such as obesity and smoking, and total QoL, underscoring the importance of risk factor modification in improving health outcomes. Patients who are aware of these risk factors are more likely to take preventive measures, such as maintaining a healthy weight and quitting smoking, which can lead to better disease management and enhanced quality of life [12]. For example, comorbid conditions, which are often related to lifestyle factors, have been found to negatively impact HRQoL in hypertensive populations, highlighting the need for comprehensive health education to address these issues [11].

Furthermore, the moderate levels of awareness about the significance of medication adherence indicate that patients comprehend certain aspects of managing hypertension, but they require additional efforts to emphasize the crucial role of adherence in blood pressure control. Non-adherence to antihypertensive medications is a common issue that can lead to poor health outcomes and lower HRQoL [9]. Previous studies have shown that educational interventions focusing on medication adherence can significantly improve adherence rates and HRQoL among hypertensive patients [8].

Finally, the moderate scores for general health and pain reported by participants are consistent with the findings of other studies that emphasize the impact of hypertension on physical functioning and overall well-being. For example, it has been found that adults with chronic conditions such as dyslipidemia, which often co-occurs with hypertension, report lower HRQoL, particularly in domains related to physical functioning and pain [13]. Addressing the physical symptoms of hypertension, such as pain, through comprehensive management strategies may help to improve HRQoL in this population. Additionally, the importance of social support in improving HRQoL among elderly individuals with chronic conditions suggests that incorporating social interventions may also benefit hypertensive patients [13].

## Conclusions

In conclusion, this study highlights the complex relationship between health awareness and HRQoL among hypertensive patients, with notable disparities between urban and rural populations. While urban patients generally demonstrated higher levels of health awareness and better HRQoL scores, rural patients faced challenges related to limited healthcare access and lower health education, particularly in areas such as lifestyle modifications and stress management. These findings underscore the need for targeted health education interventions that focus on improving awareness in rural areas to bridge these gaps. Addressing these disparities could lead to more effective hypertension management and improved quality of life, particularly for underserved rural populations.
